# Effects of Al^3+^ Substitution on Structural and Magnetic Behavior of CoFe_2_O_4_ Ferrite Nanomaterials

**DOI:** 10.3390/nano8100750

**Published:** 2018-09-21

**Authors:** Qing Lin, Yun He, Jianmei Xu, Jinpei Lin, Zeping Guo, Fang Yang

**Affiliations:** 1 College of Medical Informatics, Hainan Medical University, Haikou 571199, China; mossbauer2019@gxnu.edu.cn (Q.L.); huyang@gxnu.edu.cn (J.X.); zepingguo@gxnu.edu.cn (F.Y.); 2 Guangxi Key Laboratory of Nuclear Physics and Nuclear Technology, Guangxi Normal University, Guilin 541004, China; hy@mailbox.gxnu.edu.cn; 3 Sate Key Laboratory for Chemistry and Molecular Engineering of Medicinal Resources, Guangxi Normal University, Guilin 541004, China; 4 College of Physics and Technology, Guangxi Normal University, Guilin 541004, China; zepingguo@mailbox.gxnu.edu.cn

**Keywords:** Co-Al-Ferrite, sol-gel, Al substitution, Mössbauer, magnetic properties

## Abstract

A sol-gel autocombustion method was used to synthesize Al^3+^ ion-substituted cobalt ferrite CoAl*_x_*Fe_2−*x*_O_4_ (*x* = 0–1.5). According to X-ray diffraction analysis (XRD), cobalt ferrite was in a single cubic phase after being calcined at 1000 °C for 3 h. Moreover, the lattice constant decreased with increase in aluminum substituents. When the sample was analyzed by Scanning Electron Microscopy (SEM), we found that uniformly sized, well-crystallized grains were distributed in the sample. Furthermore, we confirmed that Al^3+^ ion-substituted cobalt ferrite underwent a transition from ferrimagnetic to superparamagnetic behavior; the superparamagnetic behavior was completely correlated with the increase in Al^3+^ ion concentration at room temperature. All these findings were observed in Mössbauer spectra. For the cobalt ferrite CoAl*_x_*Fe_2−*x*_O_4_, the coercivity and saturation magnetization decrease with an increase in aluminum content. When the annealing temperature of CoAl_0.1_Fe_1.9_O_4_ was steadily increased, the coercivity and saturation magnetization initially increased and then decreased.

## 1. Introduction

Ferrite is an important magnetic material. Cobalt ferrite is a hard ferromagnetic material, and its characteristic properties are as follows: moderate saturation magnetization (80 emu/g), high coercivity (5000 Oe), high Curie temperature T_C_ (520 °C), large anisotropy constant (2.65 × 10^5^–5.1 × 10^5^ J/m^3^) [1,2]. Cobalt ferrite has the following properties: high electromagnetic performance, large magneto-optic effect, excellent chemical stability, and excellent mechanical hardness [1,2,3]. Because cobalt ferrite is a hard ferromagnetic material, it is used as a high-density recording medium [4]. Cobalt ferrite substituted nonmagnetic Al^3+^ ions; such material has low magnetic coercivity and large resistivity. Soft ferrite is the core material in power transformers that are used in the field of electronics and telecommunication. Singhal et al. [5] used the aerosol route for substituting Fe^3+^ ions in cobalt ferrite with Al^3+^ ions. The magnetic hyperfine field decreases; the ratio of Fe^3+^(oct.)/Fe^3+^(tet.) ions increases with an increase in Al^3+^ ions. Chae et al. [6] synthesized Al*_x_*CoFe_2−*x*_O_4_ ferrite powders, and they determined magnetic properties of the sample. In Al*_x_*CoFe_2−*x*_O_4_ ferrite powders, saturation magnetization and coercive force decrease with increasing concentration of Al. In a study conducted by Kumar et al. [7], it was found that crystallite size of cobalt ferrite increased when they were doped with Al^3+^ ions. Consequently, saturation magnetization, coercive force, remnant magnetization, and magnetic anisotropy constant decreased in these doped structures. Raghavender et al. [8] investigated the dielectric properties of cobalt ferrite by doping with Al^3+^ ions. These ferrite materials exhibit low dielectric character, so they are extensively used in high-frequency applications. In this study, ferrite CoAl*_x_*Fe_2−*x*_O_4_ (*x* = 0–1.5) materials were synthesized with a sol-gel autocombustion process. The aim of this study was to determine the variation in the magnetic performance of cobalt ferrite powders, which were partially doped with nonmagnetic aluminum cations.

## 2. Experimentation

### 2.1. Sample Preparation

Cobalt ferrite powders CoAl*_x_*Fe_2−*x*_O_4_ (*x* = 0*–*1.5) were synthesized with a sol-gel autocombustion process. The raw materials of the sample were of analytical grade: Co(NO_3_)_2_·6H_2_O, Al(NO_3_)_3_·9H_2_O, Fe(NO_3_)_3_·9H_2_O, C_6_H_8_O_7_·H_2_O (citric acid), and NH_3_·H_2_O (ammonia). The molar of metal nitrates Al(NO_3_)_3_·9H_2_O was 0–0.15 mol. The molar ratio of metal nitrates to citric acid was maintained at 1:1. After weighing metal nitrates and citric acid, they were dissolved in deionized water to prepare solutions. Ammonia was added to increase the pH of the metal nitrate solution from 7 to 9. A dried gel was obtained by stirring the metal nitrate mixture in a thermostat water bath at 80 °C. Citric acid was added continuously to the dried gel. The resultant gel was dried in an oven at 120 for 2 h. The resultant powder was then burnt by igniting it in air. The dried powders were ground and sintered at specific temperatures.

### 2.2. Characterization

The structure and crystallite sizes of CoAl*_x_*Fe_2−*x*_O_4_ (*x* = 0*–*1.5) were determined by X-ray diffraction (D/max-2500V/PC, Rigaku Corporation, Tokyo, Japan) in the 2θ range of 20–70°. Micrographs were observed by scanning electron microscopy (NoVaTM Nano SEM 430, FEI Corporation, Hillsboro, OR, USA). Saturation magnetization was determined by Quantum Design MPMS series XL-7 (Quantum Design Corporation, San Diego, CA, USA). To obtain the Mössbauer spectrum, a Mössbauer spectroscope was operated in constant acceleration mode with a ^57^Co source (Fast Tec PC-mossII, FAST Corporation, Oberhaching, Bavaria, Germany).

## 3. Results and Discussion

### 3.1. X-ray Diffraction Analysis (XRD)

**Figure 1:**
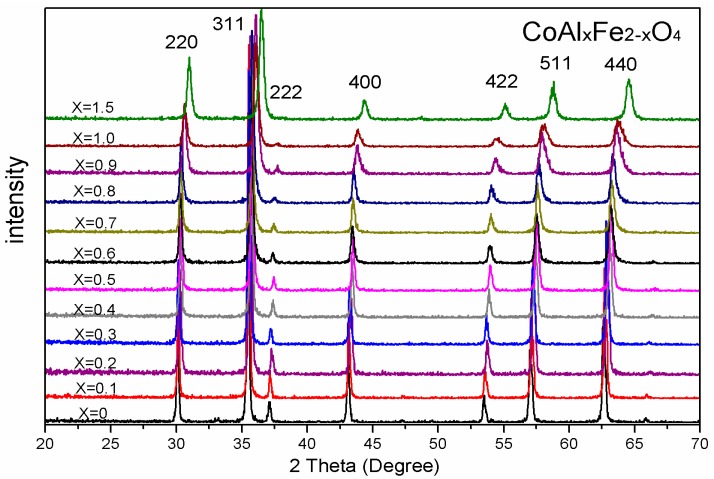
X-ray diffraction (XRD) patterns of CoAl*_x_*Fe_2−*x*_O_4_ calcined at 1000 °C.

**Figure 2:**
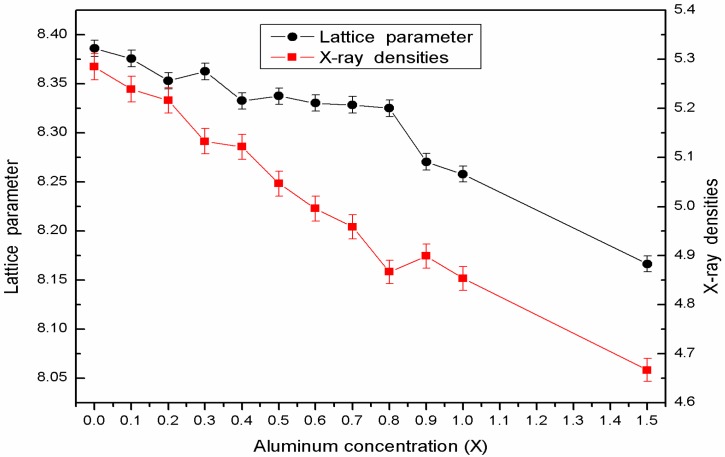
The change in the lattice parameter and X-ray densities of CoAl*_x_*Fe_2−*x*_O_4_.

Figure 1 illustrates XRD patterns for CoAl*_x_*Fe_2−*x*_O_4_ (*x* = 0–1.5) ferrites, which were calcined at 1000 °C. The XRD spectrum shows that all the samples have a single-phase structure. An impurity peak was not observed in these samples. Table 1 and Figure 2 prove that the lattice constant can be decreased by increasing the concentration of Al^3+^ ions. The decrease in lattice parameter is probably attributed to the radius of Al^3+^ ions (0.50 Å), which is smaller than Fe^3+^ ions (0.64 Å) [5,6]. X-ray density was determined from the following equation [5,8]:

(1) $$\rho_{x}=\frac{8M}{Na^{3}}$$

where a is the lattice constant; M is the relative molecular weight; and N is the Avogadro number. Table 1 and Figure 2 show that density decreases with an increase in Al^3+^ ion content. Because the atomic weight of Fe is greater than that of Al, the relative density constant decreases with increasing Al^3+^ ion substitution. X-ray density decreases under the following condition: the relative decrease in molecular mass is greater than the negligible decline in the lattice parameter. The average crystallite size decreases with an increase in the concentration of Al^3+^ ions. This phenomenon has been attributed to the size mismatch of Al^3+^ and Fe^3+^ ions, increasing strain and stress in the sample [7].

**Figure 3:**
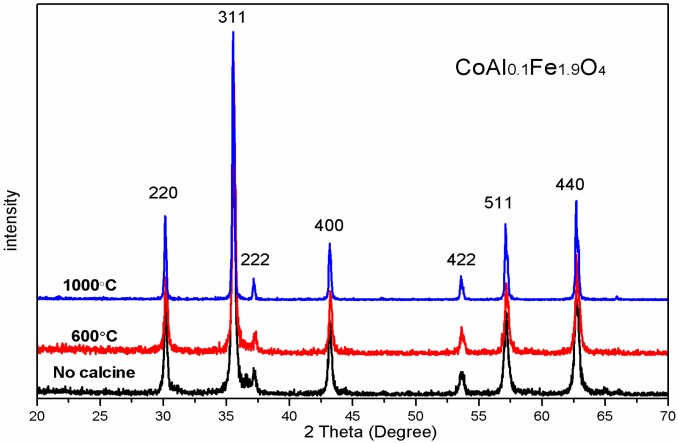
XRD patterns of ferrite CoAl_0.1_Fe_1.9_O_4_ calcined at different temperatures.

As shown in Figure 3, X-ray patterns (XRD) of CoAl_0.1_Fe_1.9_O_4_ were sintered at different temperatures. An average CoAl_0.1_Fe_1.9_O_4_ crystallite size increase by increasing the calcining temperature is observed in Table 2. All the samples were single-phase structures of spinel ferrite, which indicates the absence of an additional phase. No significant changes were observed in the lattice parameter of all samples. The average crystallite size of CoAl_0.1_Fe_1.9_O_4_ increased with an increase in calcination temperature [5].

### 3.2. Scanning Electron Microscopy (SEM)

**Figure 4:**
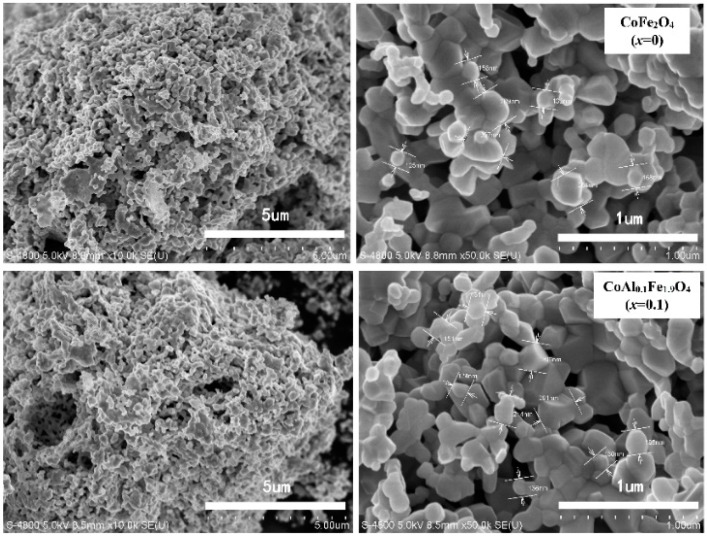
Scanning electron microscopy (SEM) micrographs of CoFe_2_O_4_ (*x* = 0) and CoAl_0.1_Fe_1.9_O_4_ (*x* = 0.1) calcined at 1000 °C.

**Figure 5:**
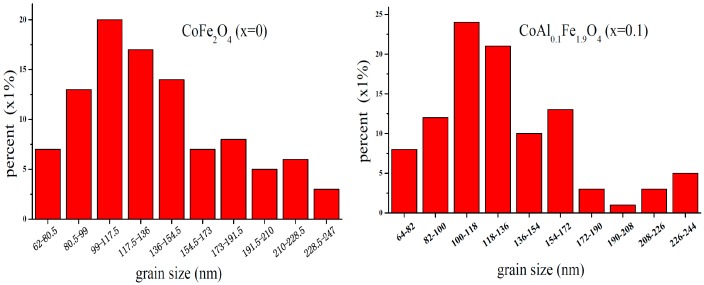
Histogram of grain-size distribution for CoFe_2_O_4_ (*x* = 0) and CoAl_0.1_Fe_1.9_O_4_ (*x* = 0.1), which were calcined at 1000 °C.

Figure 4 shows SEM micrographs of CoAl*_x_*Fe_2−*x*_O_4_ (*x* = 0, 0.1) samples, which were annealed at 1000 °C for 3 h. Uniformly-sized, well-crystallized grains of CoAl*_x_*Fe_2−*x*_O_4_ were obtained. Figure 5 illustrates the grain-size distribution of CoAl*_x_*Fe_2−*x*_O_4_ (*x* = 0, 0.1) ferrites. The average grain size of CoFe_2_O_4_ and CoAl_0.1_Fe_1.9_O_4_ was about 137.5 nm and 130.5 nm, respectively. The average grain size decreased when aluminum substituents were increased. The XRD pattern confirms that the average crystallite size tends to decrease with increasing Al content. The average grain size was greater than a nanoparticle (100 nm), and the sintering temperature of the sample was very high because grain size increased with increasing annealing temperature [9].

### 3.3. Mössbauer Spectroscopy

**Figure 6:**
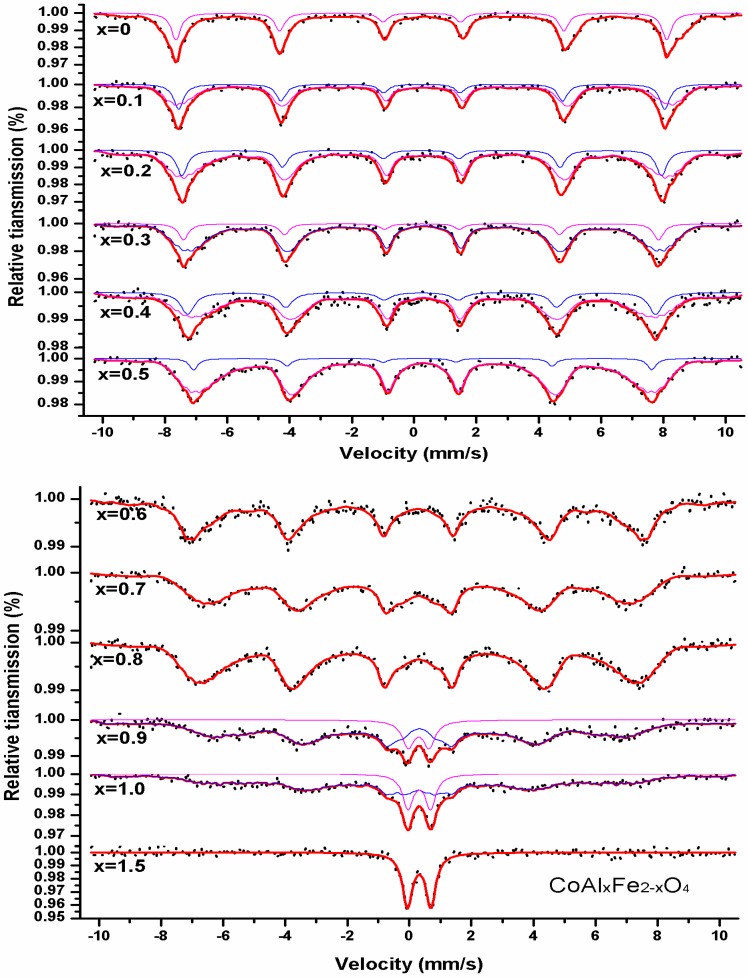
Mössbauer spectra of CoAl*_x_*Fe_2−*x*_O_4_ samples calcined at 1000 °C.

Figure 6 shows the Mösbauer spectra of CoAl*_x_*Fe_2−*x*_O_4_ acquired at room temperature. The hyperfine parameters, isomer shift (I.S.), magnetic hyperfine field (H_hf_), quadrupole shift (Q.S.), relative area (A_0_), and line width (Г), were obtained by fitted spectra using Mösswinn 3.0 software (FAST Corporation, Oberhaching, Germany), and calibration was relative to a 25 μm thick sample of high-purity alpha iron. The characteristic features of the spectra were as follows: there were two Zeeman-splitting sextets; one sextet was assigned to Fe^3+^ ion at the tetrahedral site, while the other sextet was attributed to Fe^3+^ ions at the octahedral site. This proved the ferromagnetism of the samples. The first sextet had a larger value of isomer shift, and it was assigned to octahedral B site. The second sextet had a smaller value of isomer shift, and was assigned to tetrahedral A site. Compared to the tetrahedral A-site ions, the bond separation of Fe^3+^ ions was greater in the octahedral B site of the Fe^3+^-O^2−^ complex (Table 3). This minimized the overlapping of orbits of Fe^3+^ ions at the octahedral B-site; the larger isomeric shift was attributed to smaller covalency at octahedral B site [6]. 

It is well known that the values of isomeric shift are in the range of 0.6–1.7 mm/s for Fe^2+^(S = 2) ions; the values of isomeric shift are in the range of 0.1–0.5 mm/s for Fe^3+^(S = 1/2, 3/2, 5/2) ions [10]. As shown in Table 3, the values of I.S. indicate that iron is in Fe^3+^ state. By increasing the aluminum content, the values of the magnetic hyperfine field decreased at tetrahedral A and octahedral B sites. This is because magnetic ions (Fe^3+^ ions) are substituted by nonmagnetic ions (Al^3+^ ions), affecting the supertransferred hyperfine fields [5]. For all samples, the quadrupole shift value was very small for the magnetic sextet at the A and B site. This indicates that spinel ferrites have local cubic symmetry. The spectra of CoAl*_x_*Fe_2−*x*_O_4_ (0.6 ≤ *x ≤* 0.8) included the magnetic sextet of B site; the magnetic sextet of A site vanished. This indicates that Fe^3+^ ions existed only in the octahedral B site. When the spectrum of CoAl*_x_*Fe_2−*x*_O_4_ (composition with *x* = 0.9 and 1.0) was analyzed, a single sextet and a central paramagnetic doublet were observed; this indicates relaxation effects. When the nonmagnetic Al content was increased in CoAl*_x_*Fe_2−*x*_O_4_, the samples changed into a superparamagnetic character. The behavior of the sample went from a completely magnetic state to a mixed state of magnetic and superparamagnetic order [11,12]. For samples with *x* = 1.5, Mössbauer spectra consisted only of a central doublet; this exhibits a superparamagnetic character. The central doublet was attributed to the nearest nonmagnetic neighbors of magnetically isolated Fe^3+^ ions. This leads to the deficiency of long-range magnetic ordering [13,14].

The cation distribution of CoAl*_x_*Fe_2−*x*_O_4_ ferrite can be written as follows:

(Co_β_Fe_α_Al_1−α−β_)_A_[Co_1−β_Fe_2−*x*−α_Al*_x_*_−1+α+β_]_B_O_4_ (2)

Based on the above cation distribution, the absorption-area ratio of *A* sites to *B* sites can be written as follows [12]:

(3) $$\frac{S_{A}}{S_{B}}=\frac{af_{A}}{(2-x-a)f_{B}}$$

where *f_A_* and *f_B_* are the recoil-free fractions of Fe^3+^ ions in tetrahedral *A* sites and octahedral *B* sites, respectively. The Mössbauer absorption area is proportional to the distribution of iron ions of *A* sites and *B* sites. In the current study, we assumed that *f_A_* and *f_B_* are equal [12]. Table 4 shows the cation distribution of all samples, and it was calculated using Equation (3).

### 3.4. Magnetic Analysis

**Figure 7:**
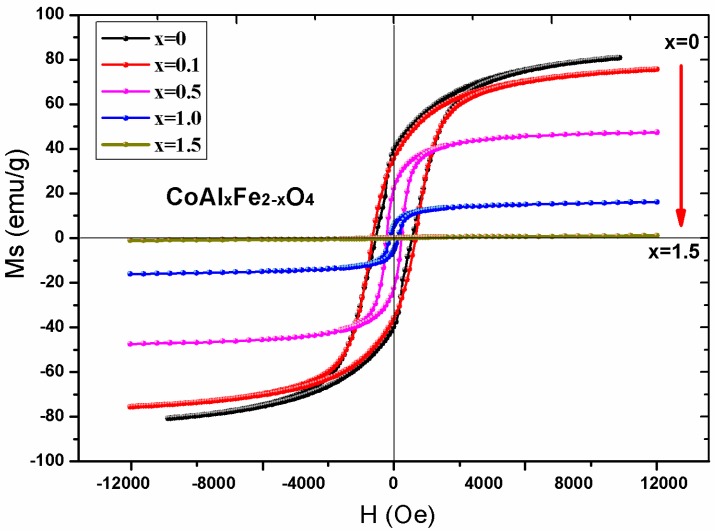
The hysteresis loops of CoAl*_x_*Fe_2−*x*_O_4_ samples calcined at 1000 °C.

**Figure 8:**
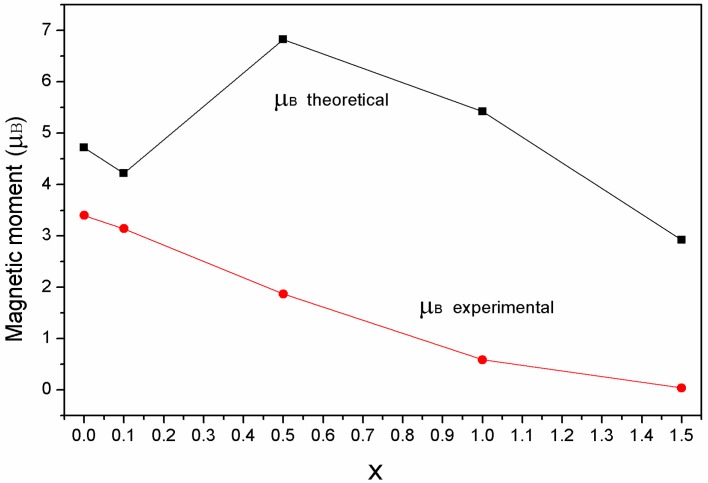
Variation in the experimental magnetic moment and calculated magnetic moment with changes in aluminum concentration.

Figure 7 illustrates the hysteresis loops of CoAl*_x_*Fe_2−*x*_O_4_ samples at room temperature. For all the samples, magnetization reached saturation when the strength of the magnetic field was 10,000 Oe. Table 5 shows that saturation magnetization decreased with an increase in Al^3+^ ion content. The saturation magnetization can be expressed with the following equation [12]:

(4) $$\sigma_{s}=\frac{5585\times n_{B}}{M}$$

where *n_B_* is the magnetic moment and M is the relative molecular mass. The relative molecular mass of CoAl*_x_*Fe_2−*x*_O_4_ decreased with an increase in Al content. The change in magnetic moment *n_B_* was determined by Néel’s theory of magnetism. The magnetic moment of Al^3+^, Co^2+^, and Fe^3+^ ions was 0 μ_B_, 3 μ_B_, and 5 μ_B_ [15,16,17], respectively. Néel’s theory of magnetism was used to develop two sublattice models, which were then used to explain cation distribution in the Mössbauer spectra (Table 4). Magnetic moment *n_B_* is expressed by Equation (5) [15,16]:

*n_B_* = *M_B_* – *M_A_* (5)

where *M_B_* and *M_A_* are magnetic moments of the *B* and *A* sublattices, respectively. Figure 8 illustrates the changes in experimental and calculated magnetic moments, with changes in Al^3+^ ion content.

Figure 8 illustrates that the experimental and calculated magnetic moment decreases with an increase in Al content (*x* ≤ 0.1). According to Equation (4), calculated saturation magnetization decreased with an increase in Al^3+^ ion substitution. The change trend of experimental and calculated saturation magnetization was similar for *x* ≤ 0.1, and there was deviation between experimental and calculated saturation magnetization, which can be attributed to the actual situation of ion distribution being more complicated than that obtained from the Mössbauer spectra. For the substituents (*x* ≥ 0.5), there was a big difference between calculated saturation magnetization and experimental saturation magnetization, and the experimental value was smaller than the calculated value for saturation magnetization [18,19,20]. This can be explained by the three-sublattice model of Yafet-Kittel (YK) [16]. It is reasonable that the spin-canting arrangement of the magnetic moment appeared on B sites of the sample when the content of nonmagnetic Al^3+^ ion substituents was too high in cobalt ferrite samples. This led to a decrease in A–B interaction and an increase in B–B interaction, which subsequently decreased magnetization.

Table 5 shows that the coercivity of CoAl*_x_*Fe_2−*x*_O_4_ decreased with an increase in Al^3+^ ion content (*x*). Based on the results of the Mössbauer spectroscopy, we inferred that Co^2+^ ions of CoFe_2_O_4_ samples were located at the tetrahedral A sites and octahedral B sites. The magnetocrystalline anisotropy is primarily attributed to Co^2+^ ions of octahedral sites, which are present in pure cobalt ferrite CoFe_2_O_4_ [7]. The electron configuration of Co^2+^ ions is 3d^7^ [21]. The anisotropy is attributed to Co^2+^ ions in the octahedral site, causing frozen orbital angular momentum and spin coupling [22]. The Al^3+^ ions elicit zero angular momentum (l = 0), which does not affect magnetic anisotropy [23,24,25]. When Al^3+^ ions were replaced with Fe^3+^ ions, the spin-orbit coupling weakened and magnetocrystalline anisotropy decreased. 

Equation (6) describes the relationship between the following parameters: coercivity *H_C_*, magnetic anisotropy *K*_1_, and saturation magnetization *M_S_* [7]:

(6) $$H_{C}=\frac{2K_{1}}{\mu_{0}M_{S}}$$

When magnetic anisotropy decreased with an increase in Al^3+^ ions, it led to a decrease in coercivity.

**Figure 9:**
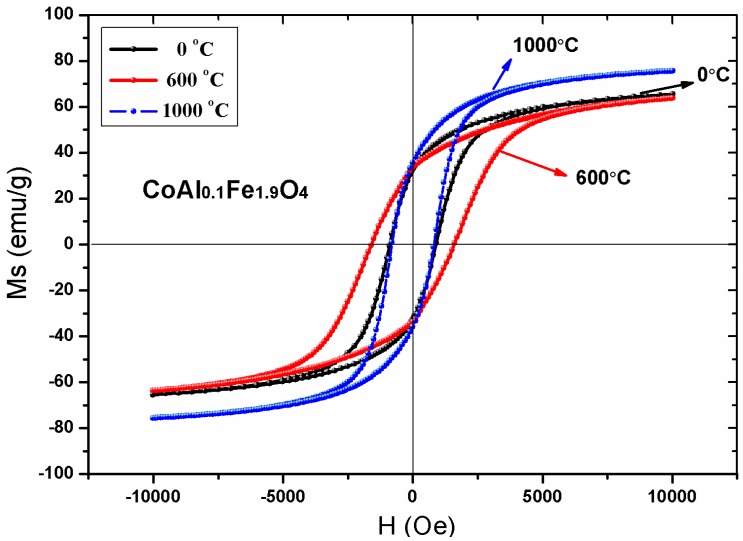
Room-temperature hysteresis curves of CoAl_0.1_Fe_1.9_O_4_ samples calcined at different temperatures.

Figure 9 shows the magnetic hysteresis curves of an unsintered CoAl_0.1_Fe_1.9_O_4_ sample at room temperature; magnetic hysteresis curves of CoAl_0.1_Fe_1.9_O_4_ sample were also obtained after sintering them at 600 °C and 1000 °C, respectively. Table 6 shows that the saturation magnetization of CoAl_0.1_Fe_1.9_O_4_ sample increased with an increase in sintering temperature; these changes were attributed to an increase in particle size [5]. There is no significant change in the saturation magnetization of the unsintered CoAl_0.1_Fe_1.9_O_4_ sample; moreover, the CoAl_0.1_Fe_1.9_O_4_ sample did not show any significant change even after being annealed at 600 °C. This confirms that the uncalcined sample has good crystallinity, which was further established by XRD.

With a steadily increasing sintering temperature, the coercivity of CoAl_0.1_Fe_1.9_O_4_ sample initially increased and then steadily decreased. This may be attributed to variation in grain size. The coercivity of the single-domain region is given by the following equation: H_C_ = g–h/D^2^. In the multidomain region, the relationship between coercivity and grain size is established by the following equation: H_C_ = (a + b)/(D). Here, ‘D’ is the diameter and ‘g, h, a, and b’ are constants of the particle [5,26]. Hence, coercivity increased with increasing grain size in the single-domain region. In the multidomain region, coercivity decreased with an increase in particle diameter [27,28]. In our study, we determined the grain size of CoAl_0.1_Fe_1.9_O_4_ samples that were calcined at different temperatures; the grain size of CoAl_0.1_Fe_1.9_O_4_ samples varied from the single-domain region to the multidomain region. With an increasing annealing temperature, the coercivity of CoAl_0.1_Fe_1.9_O_4_ sample increased initially and then decreased.

## 4. Conclusions

XRD analysis reveals the single-phase structure of CoAl*_x_*Fe_2−*x*_O_4_ samples that were calcined at 1000 °C. The lattice constant decreased when smaller Al^3+^ ions were replaced with larger Fe^3+^ ions. The XRD spectra of CoAl_0.1_Fe_1.9_O_4_ samples were obtained after sintering them at different temperatures; these samples were prepared with a sol-gel autocombustion method, so they had good crystallinity. SEM results indicate that well-crystallized particles of uniform size were present in the sample. We obtained the Mössbauer spectra of CoAl*_x_*Fe_2−*x*_O_4_ samples, which were calcined at 1000 °C. The Mössbauer spectra reveal that with an increase in aluminum concentration, CoAl*_x_*Fe_2−*x*_O_4_ samples undergo a transition from ferrimagnetic behavior to superparamagnetic behavior. Cation distribution was estimated from the Mössbauer data. The coercivity and saturation magnetization of CoAl*_x_*Fe_2−*x*_O_4_ samples decreased with an increase in Al content (*x*). The changes in saturation magnetization can be attributed to Néel’s theory and the Yafet-Kittel model. Coercivity decreased with an increase in aluminum content, which is attributed to the weakening of magnetocrystalline anisotropy. The coercivity and saturation magnetization of CoAl_0.1_Fe_1.9_O_4_ sample initially increased and then steadily decreased. Particle size increased with an increase in annealed temperature.

## Figures and Tables

**Table 1:** The XRD data of CoAl*_x_*Fe_2−*x*_O_4_ calcined at 1000 °C.

Content (*x*)	Lattice Parameter (Å)	Average Crystallite Size (Å)	Density (g/cm^3^)
0	8.38615	520	5.2847
0.1	8.37572	688	5.2392
0.2	8.35311	504	5.2161
0.3	8.36258	642	5.1328
0.4	8.33272	582	5.1216
0.5	8.33749	537	5.0470
0.6	8.33045	420	4.9959
0.7	8.32861	410	4.9583
0.8	8.32516	365	4.8666
0.9	8.27064	280	4.8992
1.0	8.25813	266	4.8534
1.5	8.16640	241	4.6668

**Table 2:** XRD data of ferrite CoAl_0.1_Fe_1.9_O_4_ calcined at different temperatures.

Temperature (℃)	Lattice Parameter (Å)	Average Crystallite Size (Å)	Density (g/cm^3^)
unsintered	8.37425	337	5.2378
600	8.36801	346	5.2537
1000	8.37572	688	5.2392

**Table 3:** Mössbauer parameters of CoAl*_x_*Fe_2−*x*_O_4_ samples, which were calcined at 1000 °C.

Content (*x*)	Component	Isomer Shift (I.S.) (mm/s)	Quadrupole Shift (Q.S.) (mm/s)	H(T)	Line Width (Γ) (mm/s)	Relative Area (A_0_) (%)
0	Sextet (A)	0.238	−0.011	48.852	0.366	28.4
	Sextet (B)	0.355	0.0004	45.889	0.338	71.6
0.1	Sextet (A)	0.245	−0.002	48.387	0.376	29.88
	Sextet (B)	0.332	−0.017	45.563	0.348	70.2
0.2	Sextet (A)	0.236	0.019	47.733	0.417	22.9
	Sextet (B)	0.334	0.001	45.360	0.348	77.1
0.3	Sextet (A)	0.236	−0.030	47.293	0.381	15.8
	Sextet (B)	0.311	−0.002	44.824	0.348	84.2
0.4	Sextet (A)	0.236	0.015	46.594	0.526	22.5
	Sextet (B)	0.307	−0.006	43.361	0.358	77.5
0.5	Sextet (A)	0.224	0.102	45.589	0.329	7.5
	Sextet (B)	0.305	−0.003	42.156	0.374	92.5
0.6	Sextet (B)	0.273	−0.048	40.664	0.424	100
0.7	Sextet (B)	0.297	−0.003	34.682	0.402	100
0.8	Sextet (B)	0.301	0.008	37.958	0.394	100
0.9	Sextet (B)	0.320	−0.003	35.164	0.341	87.6
	Double	0.302	0.670	-	0.466	12.4
1.0	Sextet (B)	0.306	−0.045	31.352	0.283	82.6
	Double	0.321	0.726	-	0.406	17.4
1.5	Double	0.318	0.752	-	0.389	100

**Table 4:** The cationic distribution of all samples, which were calcined at 1000 °C.

Sample	Cation Distribution
CoFe_2_O_4_	(Co_0.43_Fe_0.57_)_A_[Co_0.57_Fe_1.43_]_B_O_4_
CoAl_0.1_Fe_1.9_O_4_	(Co_0.43_Fe_0.57_)_A_[Co_0.57_Fe_1.33_Al_0.1_]_B_O_4_
CoAl_0.2_Fe_1.8_O_4_	(Co_0.43_Fe_0.41_Al_0.16_)_A_[Co_0.57_Fe_1.39_Al_0.04_]_B_O_4_
CoAl_0.3_Fe_1.7_O_4_	(Co_0.43_Fe_0.27_Al_0.3_)_A_[Co_0.57_Fe_1.43_]_B_O_4_
CoAl_0.4_Fe_1.6_O_4_	(Co_0.43_Fe_0.36_Al_0.21_)_A_[Co_0.57_Fe_1.27_Al_0.19_]_B_O_4_
CoAl_0.5_Fe_1.5_O_4_	(Co_0.43_Fe_0.11_Al_0.46_)_A_[Co_0.57_Fe_1.39_Al_0.04_]_B_O_4_
CoAl_0.6_Fe_1.4_O_4_	(Co_0.43_Al_0.57_)_A_[Co_0.57_Fe_1.40_Al_0.03_]_B_O_4_
CoAl_0.7_Fe_1.3_O_4_	(Co_0.43_Al_0.57_)_A_[Co_0.57_Fe_1.30_Al_0.13_]_B_O_4_
CoAl_0.8_Fe_1.2_O_4_	(Co_0.43_Al_0.57_)_A_[Co_0.57_Fe_1.20_Al_0.23_]_B_O_4_
CoAl_0.9_Fe_1.1_O_4_	(Co_0.43_Al_0.57_)_A_[Co_0.57_Fe_1.10_Al_0.33_]_B_O_4_
CoAl_1.0_Fe_1.0_O_4_	(Co_0.43_Al_0.57_)_A_[Co_0.57_Fe_1.00_Al_0.43_]_B_O_4_
CoAl_1.5_Fe_0.5_O_4_	(Co_0.43_Al_0.57_)_A_[Co_0.57_Fe_0.50_Al_0.93_]_B_O_4_

**Table 5:** Magnetic parameters of CoAl*_x_*Fe_2−*x*_O_4_ calcinated at 1000 °C obtained from hysteresis measurements.

Content (*x*)	M_s_ (emu/g)	H_c_ (Oe)	M_r_ (emu/g)	*n_B_*
0	80.89	802.77	37.15	3.40
0.1	75.66	802.76	37.75	3.14
0.5	47.43	301.11	22.54	1.87
1.0	16.13	150.56	5.45	0.59
1.5	1.06	150.38	0.10	0.04

**Table 6:** Magnetic data for CoAl_0.1_Fe_1.9_O_4_ sample calcined at different temperatures.

Temperature (°C)	M_s_ (emu/g)	H_c_ (Oe)	M_r_ (emu/g)	*n_B_*
unsintered	65.52	902.92	32.77	2.72
600	63.78	1605.13	33.53	2.65
1000	75.66	802.76	37.75	3.14
